# Secular trends of health care resource utilization and costs between Brugada syndrome and congenital long QT syndrome: A territory‐wide study

**DOI:** 10.1002/clc.24102

**Published:** 2023-07-25

**Authors:** Sharen Lee, Cheuk To Skylar Chung, Danny Radford, Oscar Hou In Chou, Teddy Tai Loy Lee, Zita Man Wai Ng, Leonardo Roever, Rajesh Rajan, George Bazoukis, Konstantinos P. Letsas, Shaoying Zeng, Fang Zhou Liu, Wing Tak Wong, Tong Liu, Gary Tse

**Affiliations:** ^1^ Cardiac Electrophysiology Unit, Cardiovascular Analytics Group PowerHealth Limited Hong Kong China; ^2^ Kent and Medway Medical School University of Kent and Canterbury Christ Church University Canterbury Kent UK; ^3^ Department of Clinical Research Federal University of Uberlandia Uberlandia Brazil; ^4^ Department of Cardiology Sabah Al Ahmed Cardiac Centre Kuwait City Kuwait; ^5^ Second Department of Cardiology Evangelismos General Hospital of Athens Athens Greece; ^6^ Arrhythmia Unit Onassis Cardiac Surgery Center Athens Greece; ^7^ Guangdong Cardiovascular Institute Guangdong Provincial People's Hospital Guangzhou China; ^8^ Department of Cardiology, Atrial Fibrillation Center, Guangdong Provincial Cardiovascular Institute, Guangdong Provincial People's Hospital Guangdong Academy of Medical Sciences Guangzhou China; ^9^ State Key Laboratory of Agrobiotechnology (CUHK), School of Life Sciences Chinese University of Hong Kong Hong Kong China; ^10^ Tianjin Key Laboratory of Ionic‐Molecular Function of Cardiovascular Disease, Department of Cardiology Second Hospital of Tianjin Medical University Tianjin China; ^11^ Division of Natural Sciences, Kent and Medway Medical School University of Kent Canterbury Kent UK

**Keywords:** Health care resource utilization

## Abstract

**Background:**

Health care resource utilization (HCRU) and costs are important metrics of health care burden, but they have rarely been explored in the setting of cardiac ion channelopathies.

**Hypothesis:**

This study tested the hypothesis that attendance‐related HCRUs and costs differed between patients with Brugada syndrome (BrS) and congenital long QT syndrome (LQTS).

**Methods:**

This was a retrospective cohort study of consecutive BrS and LQTS patients at public hospitals or clinics in Hong Kong, China. HCRUs and costs (in USD) for Accident and Emergency (A&E), inpatient, general outpatient and specialist outpatient attendances were analyzed between 2001 and 2019 at the cohort level. Comparisons were made using incidence rate ratios (IRRs [95% confidence intervals]).

**Results:**

Over the 19‐year period, 516 BrS (median age of initial presentation: 51 [interquartile range: 38−61] years, 92% male) and 134 LQTS (median age of initial presentation: 21 [9−44] years, 32% male) patients were included. Compared to LQTS patients, BrS patients had lower total costs (2 008 126 [2 007 622−2 008 629] vs. 2 343 864 [2 342 828−2 344 900]; IRR: 0.857 [0.855−0.858]), higher costs for A&E attendances (83 113 [83 048−83 177] vs. 70 604 [70 487−70 721]; IRR: 1.177 [1.165−1.189]) and general outpatient services (2,176 [2,166−2,187] vs. 921 [908−935]; IRR: 2.363 [2.187−2.552]), but lower costs for inpatient stay (1 391 624 [1 391 359−1 391 889] vs. 1 713 742 [1 713 166−1 714 319]; IRR: 0.812 [0.810−0.814]) and lower costs for specialist outpatient services (531 213 [531 049−531 376] vs. 558 597 [558268−558926]; IRR: 0.951 [0.947−0.9550]).

**Conclusions:**

Overall, BrS patients consume 14% less health care resources compared to LQTS patients in terms of attendance costs. BrS patients require more A&E and general outpatient services, but less inpatient and specialist outpatient services than LQTS patients.

## Graphical abstract

Total number of attendance in Accident and Emergency, general outpatient, general inpatient and specialist outpatient settings for Brugada syndrome and long QT syndrome from 2001 to 2019.

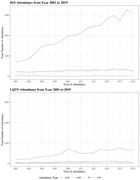

## 1 INTRODUCTION

Cardiac channelopathies can be categorized by the development of arrhythmias due to abnormalities in the function and/or structure of ion channels, resulting in syncope and sudden cardiac death (SCD).^1^ In recent years, there has been rising interest regarding the management of Long QT Syndrome (LQTS) and Brugada Syndrome (BrS). Both conditions can involve mutations in the *SCN5A* gene which encodes for the pore‐forming subunit of the cardiac sodium ion channel.^2^ LQTS is a relatively well‐documented cardiac condition, with more than 15 disease‐causing genes identified and may be seen in approximately 0.1% of the general population.^3^ In contrast, the interpretation of genetic variants in BrS is difficult, with approximately 25% of cases attributed to SCN5A mutations.^4^ Therefore, this presents a greater challenge in risk stratification and clinical management of BrS.^5^, ^6^, ^7^

However, there is little understanding of the healthcare burden of LQTS and BrS patients. The provision of genetic testing options, implantable cardioverter‐defibrillators (ICD), hospital admissions from arrhythmia‐related symptoms, and the need for specialist outpatient services follow‐up for device and arrhythmia management and monitoring are major drivers for health care expenditure.^8^, ^9^ As of now, little research has been dedicated to investigating the attendance‐related health care resource utilization (HCRU) and related costs in the setting of cardiac ion channelopathies. With increasing awareness and diagnosis of both conditions, there may be a subsequent increase in service demand, thus raising the concern for prioritization in health care interventions and specific cost‐effectiveness estimations. Without a comprehensive analysis of the cost‐effectiveness of medical technologies, this may undermine the benefit of health care policies. Locally in Hong Kong, administrative data are available on the types of attendances (such as those to Accident and Emergency (A&E) departments, inpatient admissions and their length‐of‐stay, as well as specialist and general outpatient clinics). Their official costs are published by the local government. Hence, the aim of this study is to compare the attendance‐related HCRUs and related costs between BrS and LQTS patients in Hong Kong, China.

## 2 METHODS

### 2.1 Study population

The study received ethical approval from The Joint Chinese University of Hong Kong‐New Territories East Cluster Clinical Research Ethics Committee. This territory‐wide retrospective cohort study includes patients diagnosed with BrS or LQTS between the January 1, 1997 to the December 31, 2020 in public hospitals or clinics in Hong Kong. Centralized electronic health records from the Clinical Data Analysis and Reporting System (CDARS) were used for patient identification and data extraction. This system has been used previously by our team and other teams for health care resource utilization (HCRU) and cost analysis for catecholaminergic polymorphic ventricular tachycardia,^10^ cancer patients receiving immunotherapy,^11^ and COVID‐19.^12^, ^13^ The diagnosis of LQTS and BrS was made initially by case physicians and was further verified by G.T. through documented electrocardiograms (ECGs), case notes, genetic reports and diagnostic test results in accordance with the 2017 Expert Consensus Statement for BrS.^14^

### 2.2 Clinical and electrocardiographic data collection

Our team has published previously using these LQTS and BrS cohorts for risk prediction.^15^ Baseline clinical data was extracted from the electronic health records. This included: (1) sex; (2) age of first characteristic ECG presentation and last follow‐up; (3) follow‐up duration; (4) syncope manifestation and its frequency; (5) family history of SCD and the specific ion channelopathy; (6) performance of electrophysiological study (EPS), 24‐hours Holter study, ion channelopathy‐specific genetic testing of the RYR2 gene, and the respective results; (7) presentation of sustained VT/VF and its frequency; (8) presence of other arrhythmias; (9) implantation of ICD; (10) ECG performance; (11) period between the initial presentation of characteristic ECG and the first postdiagnosis VT/VF episode; (12) initial disease manifestation (asymptomatic, syncope, VT/VF); (13) occurrence, cause and age of death. The baseline ECG was extracted at the earliest time possible after the presentation of an initial characteristic ECG pattern.

### 2.3 Statistical, HCRU and cost analyses

Categorical variables were represented as a total sum and percentage. Continuous and discrete variables were expressed as a mean and standard deviation (SD) value. Locally in Hong Kong, the various attendances to A&E, inpatient admissions, specialist or general outpatient clinic consultations are all captured by CDARS. The dates of admissions, discharges or attendances are also recorded. The official costs are published in the public domain (https://www.ha.org.hk/haho/ho/cs/238766_en.pdf). This information enabled us to calculate the HCRU and costs for A&E, inpatient, general outpatient and specialist outpatient attendances over a 19‐year period (2001−2019). Incidence rate ratios (IRRs [95% confidence intervals]) were used to conduct comparisons between the BrS and LQTS cohort. The attendance costs were calculated using unit costs in reference to the standard of the local government. Final cost values were presented in USD. Statistical significance was defined as *p* < .05. All statistical analysis was performed using R Studio (Version: 1.3.1073).

## 3 RESULTS

### 3.1 Baseline characteristics

In this study, 516 BrS patients and 134 LQTS patients were included. The average age at first presentation was much younger for the LQTS cohort compared to the BrS cohort (27.6 ± 23.8 vs. 49.9 ± 16.2). In addition, the LQTS cohort had a greater percentage of females (67.9% vs. 7.6%), as well as more patients with a family history of the disease (43.3% vs. 3.1%) and VF/SCD (14.9% vs. 7.9%) compared to the BrS cohort. The number of genetic tests performed was also higher in the LQTS cohort compared to the BrS cohort (84 vs. 51). Interestingly, the BrS cohort performed significantly more EPS (112 vs. 6) and had a greater proportion of induced VT/VF (14.7% vs. 3.0%). In regard to the baseline ECG characteristics, BrS patients had overall longer PR interval (169.5 ± 29.0 vs. 161.8 ± 29.8) and P‐wave duration (114.7 ± 18.1 vs. 105.1 ± 17.5) but shorter QTc interval (368.9 ± 42.4 vs. 488.5 ± 44.4) compared to the LQTS cohort. The baseline characteristics comparing the LQTS and BrS cohort are summarized in Table 1.

**Table 1:** Baseline characteristics of the study cohort.

Variable	LQTS (*n* = 134)	BrS (*n* = 516)
Clinical characteristics		
Female	91 (67.9)	39 (7.6)
Age at first presentation	27.6 ± 23.8	49.9 ± 16.2
Family history of LQTS/BrS	58 (43.3)	16 (3.1)
Family history of VF/SCD	20 (14.9)	41 (7.9)
Syncope	68 (54.4)	222 (43.0)
Spontaneous VT/VF in follow‐up	51 (38.1)	80 (15.5)
Initial VT/VF	34 (25.4)	42 (8.1)
Treadmill performed	50 (37.3)	63 (12.2)
EPS	6 (4.5)	112 (21.7)
*Induced VT/VF under EPS*	4 (3.0)	76 (14.7)
ICD	51 (38.1)	136 (26.4)
Genetic test	84 (62.7)	51 (9.9)
Baseline ECG characteristics		
Heart rate (bpm)	76.7 ± 23.4	80.9 ± 20.0
P‐wave duration (ms)	105.1 ± 17.5	114.7 ± 18.1
PR interval (ms)	161.8 ± 29.8	169.5 ± 29.0
QRS interval (ms)	95.4 ± 21.9	106.4 ± 22.7
QT interval (ms)	444.2 ± 71.8	416.5 ± 33.2
QTc Interval (ms)	488.5 ± 44.4	368.9 ± 42.4
P axis	54.9 ± 40.4	61.3 ± 22.3
QRS axis	55.1 ± 56.9	58.7 ± 39.6
T axis	52.9 ± 54.9	54.3 ± 26.0
R wave in lead V5	1.2 ± 0.7	1.5 ± 0.6
S wave in lead V1	0.7 ± 0.4	0.6 ± 0.3

*Note*: Categorical and continuous variables were compared between LQTS and BrS patients.

Abbreviations: BrS, Brugada syndrome; LQTS, long QT syndrome.

### 3.2 HCRU and cost analysis

The total number of attendances for A&E, inpatient, general and specialist outpatient setting in the cohort is as follows: 5154, 4140, 373 and 34 049 for the BrS cohort and 1137, 1285, 41, 9298 for the LQTS cohort. Both cohorts demonstrated the highest number of attendance in the specialist outpatient settings, however the BrS cohort had a greater overall number of attendance compared to the LQTS cohort (43 716 vs. 11 761). In addition, the attendance number of inpatient length of stays of the BrS cohort were also significantly higher than the LQTS cohort (20 813 vs. 6656). The attendance and costs of the BrS and LQTS cohort are shown in Table 2.

**Table 2:** Cohort‐level attendance, length of stay and costs for BrS and LQTS patients.

Hospital setting	Variable	Attendance		Length of stay		Cost (USD)	
		BrS	LQTS	BrS	LQTS	BrS	LQTS
Accident and Emergency	Total	5154	1137	‐	‐	814 835	179 757
	Total (million)	0.01	0.00	‐	‐	1	0
	Total per patient	9.99	8.49	‐	‐	1579	1341
	Total per patient‐year	0.53	0.45	‐	‐	83	71
	Total per patient‐year LCI	0.51	0.42	‐	‐	83	70
	Total per patient‐year UCI	0.54	0.47	‐	‐	83	71
Inpatient	Total	4140	1285	20 813.00	6656.00	13 643 483	4 363 188
	Total (million)	0.00	0.00	0.02	0.01	14	4
	Total per patient	8.02	9.59	40.34	49.67	26 441	32 561
	Total per patient‐year	0.42	0.50	2.12	2.61	1392	1714
	Total per patient‐year LCI	0.41	0.48	2.09	2.55	1391	1713
	Total per patient‐year UCI	0.44	0.53	2.15	2.68	1392	1714
General outpatient	Total	373	41	‐	‐	21 335	2345
	Total (million)	0.00	0.00	‐	‐	0	0
	Total per patient	0.72	0.31	‐	‐	41	18
	Total per patient‐year	0.04	0.02	‐	‐	2	1
	Total per patient‐year LCI	0.03	0.01	‐	‐	2	1
	Total per patient‐year UCI	0.04	0.02	‐	‐	2	1
Specialist outpatient	Total	34 049	9298	‐	‐	5 208 009	1 422 188
	Total (million)	0.03	0.01	‐	‐	5	1
	Total per patient	65.99	69.39	‐	‐	10 093	10 613
	Total per patient‐year	3.47	3.65	‐	‐	531	559
	Total per patient‐year LCI	3.44	3.58	‐	‐	531	558
	Total per patient‐year UCI	3.51	3.73	‐	‐	531	559
All	Total	43 716	11 761	‐	‐	19 687 663	5 967 478
	Total (million)	0.04	0.01	‐	‐	20	6
	Total per patient	84.72	87.77	‐	‐	38 154	44 533
	Total per patient‐year	4.46	4.62	‐	‐	2008	2344
	Total per patient‐year LCI	4.39	4.49	‐	‐	2008	2343
	Total per patient‐year UCI	4.53	4.76	‐	‐	2009	2345

*Note*: Median (lower and upper 95% confidence intervals) values are presented. Costs shown are in US dollars.

Abbreviations: BrS, Brugada syndrome; LQTS, long QT syndrome.

**Figure 1:**
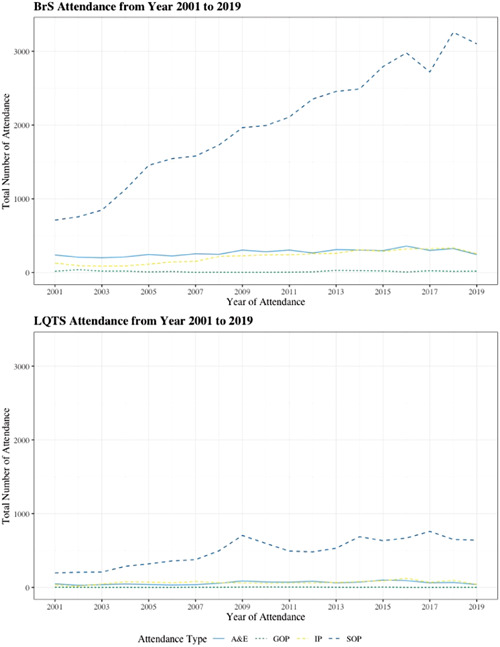
Total number of attendance in A&E, general outpatient, general inpatient and specialist outpatient settings for BrS and LQTS from 2001 to 2019. A&E, Accident and Emergency BrS, Brugada syndrome; LQTS, long QT syndrome.

Between 2001 and 2019, the number of attendances in specialist settings was highest among the four settings for both BrS and LQTS cohorts. The data also shows a general increase in the number of attendances in specialist settings during this time period. Additionally, the number of attendances in specialist A&E, general outpatient and general inpatient settings were consistently higher in the BrS cohort throughout the time period, with the exception of 2009 and 2010, where the number of attendances in general outpatient setting was slightly higher in the LQTS cohort. The results of the total number of attendances in A&E, general outpatient, general inpatient, and specialist outpatient settings for the BrS and LQTS cohorts from 2001 to 2019 are shown in Figure 1.

In comparison to LQTS patients, BrS patients had lower overall costs (2 008 126 [2 007 622−2 008 629] vs. 2 343 864 [2 342 828−2 344 900]; IRR: 0.857 [0.855−0.858]) (Table 3). To corroborate BrS patients had higher costs for A&E attendances (83 113 [83 048−83 177] vs. 70 604 [70 487−70 721]; IRR: 1.177 [1.165−1.189]) and general outpatient services (2176 [2166−2187] vs. 921 [908−935]; IRR: 2.363 [2.187−2.552]) relative to LQTS patients. In contrast, LQTS patients had higher costs for inpatient stay (1 713 742 [1 713 166−1 714 319] vs. 1 391 624 [1 391 359‐1 391 889]; IRR: 0.812 [0.810−0.814]) and slightly higher costs for specialist outpatient services (558 597 [558 268−558 926]; vs. 531 213 [531 049−531 376]; IRR: 0.951 [0.947−0.9550]) compared to BrS patients.

**Table 3:** Cohort‐level health care utilization and costs for BrS and LQTS patients.

Attendance type	Total all‐cause attendances per 1000 patient‐years		Total all‐cause costs ($) per 1000 patient‐years		Incidence rate ratios (95% confidence intervals)
	BrS	LQTS	BrS	LQTS	BrS versus LQTS
Accident and Emergency	525.7 (511.5−540.3)	446.7 (421.0−473.3)	83 113 (83 048−83 177)	70 604 (70 487−70 721)	1.177 (1.165−1.189)
Inpatient	422.3 (409.5−435.3)	504.7 (477.5−533.1)	1 391 624 (1 391 359−1 391 889)	1 713 742 (1 713 166−1 714 319)	0.812 (0.810−0.814)
General Outpatient	38.1 (34.3−42.1)	16.1 (11.6−21.9)	2176 (2166−2187)	921 (908−935)	2.363 (2.187−2.552)
Specialist Outpatient	3473.0 (3436.2−3510.1)	3652.0 (3578.2−3727.0)	531 213 (531 049−531 376)	558 597 (558 268−558 926)	0.951 (0.947−0.955)
All	4459.0 (4391.4−4527.8)	4619.4 (4488.2−4755.2)	2 008 126 (2 007 622−2 008 629)	2 343 864 (2 342 828−2 344 900)	0.857 (0.855−0.858)

*Note*: Median (lower and upper 95% confidence intervals) values are presented. Costs shown are in US dollars.

## 4 DISCUSSION

This is the first territory‐wide cohort study in Hong Kong to compare the attendance‐related health care costs of LQTS and BrS patients. The major findings of this study is as follows: (1) BrS patients consume 14% less health care resources compared to LQTS patients; (2) BrS patients require more services from A&E and general outpatient setting; (3) LQTS patients require more services from inpatient and special outpatient setting.

The present study suggests that there are drastic differences in the health care burden of the two cardiac channelopathies. Due to the lower caseload of LQTS compared to BrS in Hong Kong,^16^, ^17^, ^18^ a greater percentage of patients had to undergo more cardiological examinations and consultations at a clinical genetics department. It may be argued that genetic testing plays a greater role in the diagnosis of LQTS relative to BrS because current genetic knowledge of LQTS is more advanced and the disease is more likely to have a genetic origin.^19^ Even for patients with inconclusive clinical scores, the current referral practice for LQTS often entails for aggressive treatment including rigorous restrictions on the patient's lifestyle and primary ICD implantation.^20^ Resultantly, this may warrant unnecessary expenditure on patients who are at low‐risk or have no risk of LQTS.

This notwithstanding, the family members of the LQTS patient are often included in the confirmatory testing process to screen for concealed or preclinical LQTS.^21^ Through early identification of familial LQTS, this will allow patients to receive timely secondary and tertiary prevention. Subsequently, this may lead to the increase in financial costs and anxiety for the patient. The 5‐gene version of the FAMILION LQTS test costs approximately $5400 per index case and $900 per family member as a confirmatory test.^22^ Although newer technologies demonstrate great potential in reducing costs of intervention and detection of new mutations,^23^ the lack of competition minimizes the commercial incentive in finding new alternatives. However, current prices for diagnostic assessments were still significantly less expensive compared to previous genetic tests without genetic testing strategies.^24^ Despite advancements made in the understanding of LQTS genetics, the distinction between pathogenic and benign variants in LQTS‐susceptibility genes remains challenging for physicians.^25^ Hence, this also warrants the need for further refinement of the clinical interpretation of LQTS to reduce the number of false positive and familial LQTS patients, and ultimately health care costs.^26^ Furthermore, it is also crucial to consider the relevant health care policies and insurance regulations of individual hospitals. Therefore, this may explain the health care cost discrepancies between the LQTS and BrS cohort.

### 4.1 Strengths and limitations

Several major strengths were demonstrated in this study: (1) costs were estimated using standardized unit costs across extended follow‐up periods; (2) the sampling of one of the largest cardiac channelopathies cohorts available enhances the reliability of study findings; (3) the use of a public, comprehensive electronic health record system from the city, incorporating attendances from 43 hospitals and their associated outpatient and ambulatory care facilities.

Several limitations should also be noted. The retrospective observational nature of this study suggests that results may be prone to coding errors, under‐coding or missing data, resulting in information and selection bias. However, as the majority of patients were closely followed‐up through annual consultations, the bias was amended with detailed follow‐up and patient documentation. Although the database used already documents one of the largest cohorts of cardiac channelopathies in Asia, the sample size is still small compared to other cardiac diseases, especially the LQTS cohort. Consequently, this limits the validity of study findings. This is due to the fact that the prevalence of BrS and LQTS is low relative to other cardiac diseases in Hong Kong. It is prudent to recognize that our cost analyses require additional external validation in future studies.

## 5 CONCLUSIONS

In conclusion, major differences in economic burden between BrS and LQTS patients were identified in this study. These findings can offer novel insight into the financial management of clinical interventions and optimization of health care policies surrounding BrS and LQTS. However, it is imperative that further research is conducted to extend the costs analysis amongst subgroups in both cohorts.

## AUTHOR CONTRIBUTIONS

Sharen Lee and Cheuk To Skylar Chung—statistical analysis, data analysis, data interpretation, cost analysis, manuscript drafting. Danny Radford, Oscar Hou In Chou, Tong Liu, Zita Man Wai Ng, Konstantinos P Letsas, Leonardo Roever, Rajesh Rajan, George Bazoukis, Konstantinos P. Letsas, Shaoying Zeng, Fang Zhou Liu, Wing Tak Wong, Tong Liu—data analysis, manuscript revision. Gary Tse—data acquisition, database building, cost analysis, study conception, statistical analysis, manuscript drafting, and manuscript revision

## CONFLICT OF INTEREST STATEMENT

The authors declare no conflict of interest.

## Data Availability

Deidentified data is available from the corresponding author upon reasonable request.
